# *In silico* and *in vitro* studies reveal complement system drives coagulation cascade in SARS-CoV-2 pathogenesis

**DOI:** 10.1016/j.csbj.2020.11.005

**Published:** 2020-11-11

**Authors:** Ritudhwaj Tiwari, Anurag R. Mishra, Flora Mikaeloff, Soham Gupta, Ali Mirazimi, Siddappa N. Byrareddy, Ujjwal Neogi, Debasis Nayak

**Affiliations:** a Discipline of Biosciences and Biomedical Engineering, Indian Institute of Technology Indore, Indore, MP, India; b Division of Clinical Microbiology, Department of Laboratory Medicine, Karolinska Institutet, Stockholm, Sweden; c Public Health Agency of Sweden, Solna, Sweden; d National Veterinary Institute, Uppsala, Sweden; e Department of Pharmacology and Experimental Neuroscience, University of Nebraska Medical Center, Omaha, NE, USA; f Department of Molecular Microbiology and Immunology and the Bond Life Science Center, University of Missouri, Columbia, MO 65211, USA

**Keywords:** SARS-CoV-2, Thrombosis, MAPK1/MAPK3, ARDS, Cytokines storm, Neutrophil degranulation

## Abstract

The emergence and continued spread of SARS-CoV-2 have resulted in a public health emergency across the globe. The lack of knowledge on the precise mechanism of viral pathogenesis is impeding medical intervention. In this study, we have taken both *in silico* and *in vitro* experimental approaches to unravel the mechanism of viral pathogenesis associated with complement and coagulation pathways. Based on the structural similarities of viral and host proteins, we initially generated a protein-protein interactome profile. Further computational analysis combined with Gene Ontology (GO) analysis and KEGG pathway analysis predicted key annotated pathways associated with viral pathogenesis. These include MAPK signaling, complement, and coagulation cascades, endocytosis, PD-L1 expression, PD-1 checkpoint pathway in cancer and C-type lectin receptor signaling pathways. Degree centrality analysis pinned down to MAPK1, MAPK3, AKT1, and SRC are crucial drivers of signaling pathways and often overlap with the associated pathways. Most strikingly, the complement and coagulation cascade and platelet activation pathways are interconnected, presumably directing thrombotic activity observed in severe or critical cases of COVID-19. This is complemented by *in vitro* studies of Huh7 cell infection and analysis of the transcriptome and proteomic profile of gene candidates during viral infection. The most known candidates associated with complement and coagulation cascade signaling by KEGG pathway analysis showed significant up-regulated fold change during viral infection. Collectively both *in silico* and *in vitro* studies suggest complement and coagulation cascade signaling are a mechanism for intravascular coagulation, thrombotic changes, and associated complications in severe COVID-19 patients.

## Highlights

• MAPK1, MAPK3, AKT1, and SRC proteins are the critical drivers of signaling pathways and often overlap with the associated pathways during SARS-CoV-2 infection.
• Viral proteins interact with MAVS, TRAF6, IRAK1, IRF3, and IRF7 and inhibit IFN-I and ISGs production.
• TP53, TNF, MAPK3 proteins in Cytokines storm and VAMP8, ITGAM, and STOM in Neutrophils degranulation are regulatory proteins associated with the ARDS.
• Proteomics data showed 28 candidates associated with complement and coagulation cascade during SARS-CoV-2 infection.
• Our study suggests that therapeutic targeting of the downstream proteins of the complement system can mitigate SARS-CoV-2 pathogenesis.

## Graphical abstract

**Figure:**
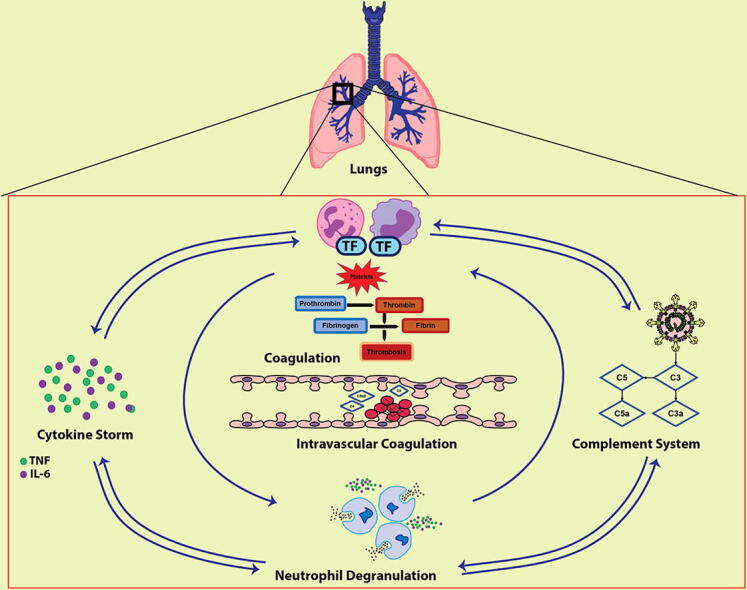

## 1 Introduction

Coronavirus disease 2019 (COVID-19) is a respiratory disease that primarily manifests with pneumonia-like symptoms, gastrointestinal symptoms and is occasionally associated with multiorgan failure [1], [2]. The causative agent SARS-CoV-2 is an enveloped positive-sense, single-stranded RNA betacoronavirus, which belongs to the Coronaviridae family. Six human coronaviruses (HCoVs) were previously identified. However, over the past two decades, highly pathogenic HCoVs have emerged. These include SARS-CoV in 2002 with a mortality rate of 10% and MERS-CoV emerged in 2012 with a case fatality rate of 36% [3], [4]. Although the overall mortality rate due to the current SARS-CoV-2 is relatively lower, the virus seemingly spreads more efficiently, thus making it far more challenging to contain and increasing its pandemic potential. Despite continuous research activities, efficacious vaccines or antivirals may be months or even years away. To understand the pathophysiology of the virus and develop new therapeutics, it is necessary to develop a broad understanding of host-pathogen interactions; like how SARS-CoV-2 uses the host machinery during infection, and apply this information towards developing both new drugs and repurposing existing drugs.

In this study, we utilized *in silico* methods for predicting the interactions between SARS-CoV-2 and the host proteins followed by transcriptomics and proteomics analysis of the SARS-CoV-2 infected Huh7 cell-line. The approach is based on protein structural similarity. We predicted an interaction map for SARS-CoV-2 cellular co-localization information by mining previously published data sets of coronaviruses and other respiratory disease-causing RNA viruses [5], [6], [7]. Collectively, with the knowledge of protein–protein interactions of SARS-CoV-2, these predictions provide an additional platform for a better understanding of viral pathogenesis and identification of potential clinical targets. Gene enrichment analysis of these interacting host proteins advocates that cytokine storm and neutrophil degranulation drive acute respiratory disease syndrome (ARDS) in SARS-CoV-2 patients. Most strikingly, the complement and coagulation cascade are interconnected and potentially crucial drivers of the innate immune response against SARS-CoV-2. This prediction was further validated by the KEGG pathway in gene set enrichment analysis of combined transcriptomics and proteomics data sets from *in vitro* SARS-CoV-2 infections in cell lines as reported earlier [8]. Thus the current study helps us to understand the molecular mechanism of the SARS-CoV-2 infection and its role in pathogenesis through the combined activation of cytokine storm, neutrophil degranulation, and the complement system.

## 2 Materials and methods

### 2.1 Data sources

The crystal and cryo-EM structures of SARS-CoV-2 [main protease (PDB ID:5R7Y), Spike glycoproteins (PDB ID: 6VSB, 6VXX), HR2 Domain (PDB ID:6LVN), NSP15 (PDB ID:6VWW), NSP3 (PDB ID:6W02) and NSP9 (PDB ID: 6W4B)] were obtained from RCSB Protein Data Bank (PDB). The rest of the SARS-CoV-2 protein structures (NSP1, NSP2, NSP4, NSP6, NSP7, NSP8, NSP10, NSP12, NSP13, NSP14, NSP16, ORF3a, E, M, ORF6, ORF7a, ORF8, N, ORF10) were curated from Zhang Lab, which modeled these structures by using I-TASSER [9]. Each of the structures for SARS-CoV-2 proteins were compared with proteins of known structure for structural similarities using the DaliLite v.5 webservers [10]. The PDB codes obtained from Dali were mapped to their corresponding Uniprot ID and gene Name by DAVID Gene ID Conversion or Uniprot ID mapping [11], [12]. Gene Ontology (GO) analysis of these proteins was performed using the g: Profiler tool and Reactome database. Their interaction networks and degree centrality calculations were annotated using the STRING and CytoNCA tool in Cytoscape [13], [14], [15], [16].

### 2.2 Determination of structural similarities between SARS-CoV-2 and human proteins

We determined the structural similarities among SARS-CoV-2 and human proteins from DaliLite v. 5 webservers [10]. The Dali server compares 3D structural coordinates of two PDB entries by an alignment of alpha carbon distance matrices, allowing for differences in domain order, and finally produces a structural similarity score. In the current study, we submitted each SARS-CoV-2 protein (PDB and Zhang Lab) into the Dali web server that searched against the entire PDB dataset for structurally-similar proteins with a z-score above 2.0. As a result, we retrieved all the proteins having structures similar to SARS-CoV-2 proteins available in the PDB database. From these results, we then filtered to only include those structures pertaining to human host. These human SARS-CoV-2-similar proteins are hence referred to as “hSARS-CoV-2 similar” proteins.

### 2.3 Interaction prediction

To predict the human endogenous proteins interacting with the SARS-CoV-2 proteins, we investigated the target proteins interacting with the hSARS-CoV-2 similar proteins during various cellular processes. We found known interactions between hSARS-CoV-2 similar proteins and target human endogenous proteins using data from the BIOGRID, HPRD, and MINT database [17], [18], [19]. These datasets are sourced from literature-curated interactions among human proteins. It is presumed that these cellular proteins, which are known to interact with human protein (similar to SARS-CoV-2 structures), might also interact with SARS-CoV-2 proteins due to their structural similarity.

### 2.4 Cellular compartmentalization (CC) and gene enrichment analysis

The assumed host protein interactors of the SARS-CoV-2 proteome were shortlisted based on their functionality and cellular localization. The interactor host proteins were primarily annotated on the basis of protein localization, since theoretically, the two proteins must share at least one cellular compartment for direct communication among them. In our study, cellular compartments were chosen based on published literature and availability in the UniProt database, suggesting the localization of viral proteins in the host cell. Classified proteins from CC study were then submitted to the g:Profiler and Reactome database to retrieve the list of terms enriched with these proteins and the biological pathways, including them [13], [15].

### 2.5 Validation of predictions

As Dali might contain multiple PDB structures of the same protein, repetition in the interaction predictions could surface. Some SARS-CoV-2 proteins have multiple PDB structures resulting in similar repetitive interaction predictions. Hence, prediction count based on a pair of human Uniprot accessions and SARS-CoV-2 protein name was set as “single” score. The predicted interactions supported by the data obtained from experimental studies carried out for SARS-CoV-2 and datasets from related RNA viruses obtained from our VHFIDB database (www.vhfidb.com) create a very comprehensive and manually curated database, containing 9921 host factors information for 72 viral species.

### 2.6 Complement and coagulation cascades pathway in SARS-CoV-2 infection model

We re-analyzed the proteo-transcriptomics data of SARS-CoV-2 infected (1 MOI) Huh7 cells to identify the temporal changes resulted from infection over time [8]. Briefly, Huh7 cells infected with SARS-CoV-2 were collected at 24, 48, and 72 h in triplicates. Transcripts and protein abundance were quantified using RNA-seq and LC-MS/MS methods. To identify the relative genes changes, temporal differential abundance analysis was performed using a univariate time series model from the R package LIMMA. In the LIMMA design matrix, separated coefficients were associated with time and replicates to extract the difference as a contrast. Moderated paired *t*-test using LIMMA with adjustment for replicates was used. Benjamini-Hochberg (BH) adjustment was applied, and only proteins and transcripts with adjusted p values <0.05 were selected. Eighty-five genes associated with KEGG pathway “Complement and coagulation cascades – Homo sapiens” were retrieved from the KEGG database (https://www.genome.jp/kegg-bin). The proteins and transcripts associated with complement and coagulation networks were created separately with Cytoscape ver 3.6.1. For each node, fold change and q-value from LIMMA were added to the network template file. Edges between nodes were taken from the string database (https://string-db.org/) with high evidence (interaction score >700), and interactions retrieved from experiments and databases only. Nodes refer to connected objects in the network and edges to the connections between nodes. Heatmap was created using R package heatmap 2.

## 3 Results

### 3.1 Identification of SARS-CoV-2-similar human proteins (hSARS-CoV-2) and hSARS-CoV-2 host interaction

To identify the list of human host proteins having structural similarity to SARS-CoV-2 proteins, we employed a previously established protocol [20] by collecting the available PDB structures of viral proteins and predicted structures from the Zhang lab [9]. The viral protein structure was then submitted to DaliLite v.5 web servers for predicting structure similarity to human proteins. We refer to these identified human proteins bearing a domain of high structural similarity to the SARS-CoV-2 protein as “SARS-CoV-2 -similar”. Next, we determined the known interactions for these SARS-CoV-2 -similar human (hSARS-CoV-2) proteins. The structural similarity analysis identified 3735 human proteins (hSARS-CoV-2) similar to the 16 SARS-CoV-2 proteins. Next, we identified all possible interaction partners for these proteins and identified the interacting partners of the 16 proteins of SARS-CoV-2 from all interacting partners of the hSARS-CoV-2 similar proteins downloaded from three different databases (HPRD, BIOGRID, and MINT). A total of 57,359 unique interaction partners were identified for these 16 SARS-CoV-2 proteins, involving 12,872 unique human interacting proteins (Supplementary Table 1). To further enrich the protein pool, we used two different kinds of filters (cellular compartment analysis (CC), and literature analysis). First, we used GO cellular compartment (CC) annotation to refine and filter the candidates using the cellular localization pattern. This exercise reduced the number of unique interaction partners to 19,047, with 6876 unique proteins (Supplementary Table 2). Later we curated the data by analyzing the RNA virus dataset, filtered, and obtained 5903 unique interaction partners with 2647 unique proteins. This information was subsequently used for the functional pathway analysis and for determining the role played by genes in associated pathways.

We compared our predictions with a previously published study by Gordon et al.. Experimental data sets published in this study showed SARS-CoV-2 and human proteins resulted in 332 unique interactions. When we aligned our interacting protein list with that from Gordon et al., we noticed around 91% overlap with our primary interacting list and about 54% genes with our CC and literature filtered list (Fig. S1).

### 3.2 Gene enrichment analysis of SARS-CoV-2 interactor proteins

**Fig. 1:**
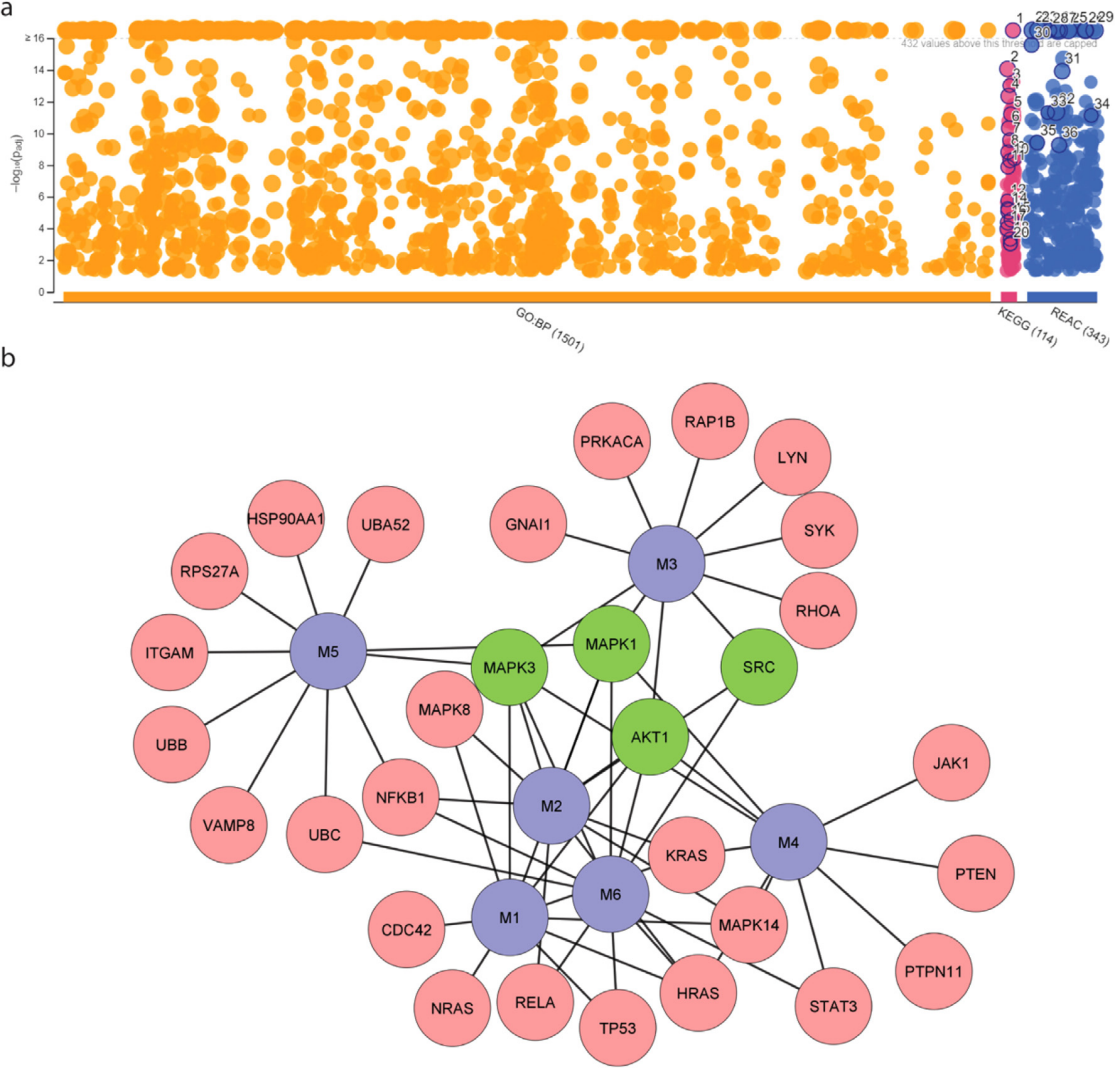
GOSt multi-query Manhattan plot and Degree centrality analysis of overlapping proteins in the profoundly enriched pathways. (a) g: GOSt multi-query Manhattan plot shows significantly enriched GO: BP, KEGG terms, and Reactome enhanced data for SARS CoV-2 interacting proteins. (b) Network analysis based on the input pathways, showing overlapping proteins between 6 highly enriched pathways. Of these, the subnetwork shows MAPK1, MAPK3, AKT1, and SRC proteins are connected maximally. Abbreviations: M1: MAPK signaling pathway, M2: C-type lectin receptor signaling pathway, M3: Platelet activation, M4: PD-L1 expression, and PD-1 checkpoint pathway in cancer, M5: Innate Immune System, M6: Cytokine Signaling in Immune system.

Next, we performed gene enrichment analysis by using the g: GOSt tool of g: Profiler. g: GOSt is the core tool for performing functional enrichment analysis on the input gene list [15]. It mapped a handler-provided list of genes to find relevant information sources and detect statistically significant enriched biological processes and pathways. At first, we uploaded the SARS CoV-2 interactors protein lists in. g: GOSt tools and manually selected GO: biological process (GO: BP), KEGG pathways, and Reactome database in data source option and set threshold value at 0.05. Finally, we got g: GOSt multi-query Manhattan plot, which shows significantly enriched GO: BP, KEGG terms and Reactome enhanced data (Fig. 1 A, Fig. S2). Subsequent Reactome dataset analysis revealed that SARS-CoV-2 interactors proteins are enriched with the terms of biomedical pathways. Similarly, the KEGG pathway showed most of the genes associated with salmonella infection, MAPK signaling pathway, complement, and coagulation cascades, endocytosis, PD-L1 expression, and PD-1 checkpoint pathway in cancer and C-type lectin receptor signaling pathway. To check the essential proteins of these signaling pathways and their overlapping genes, we performed the degree centrality analysis of genes associated with highly enriched pathways using the CytoNCA tool of Cytoscape. The degree centrality analysis revealed that the MAPK1, MAPK3, AKT1, and SRC proteins play crucial role in six highly relevant biomedical pathways. These include (i) cytokine signaling in the immune system, (ii) MAPK signaling pathway, (iii) PD-L1 expression, and PD-1 checkpoint pathway in cancer, (iv) platelet activation, (v) Innate immune system, and (vi) C-type lectin receptor signaling pathways (Fig. 1B).

### 3.3 MAPK is predicted to be the key player in the innate immune response

Coronavirus can evade the host innate immune response by interacting with key components of signaling pathways. The GO enrichment analysis of predicted candidates showed 369 host genes associated with the innate immune system to interact with SARS-CoV-2 proteins (Supplementary Table 3). *In silico* analysis revealed that viral proteins like S, NSP13, NSP15, NSP7, NSP8, NSP 5, and NSP4 interact with mitochondrial antiviral-signaling protein (MAVS), an important intermediate of RIG-I signaling-mediated IFN-I production. This interaction of the viral proteins with MAVS can inhibit IFN-I production. For example, blocking CARD-CARD domain interaction between RIG-I and MAVS, thereby promotes proteasomal degradation of MAVS or interferes with the recruitment of tumor necrosis factor receptor-associated factor (TRAF) family proteins [21]. Notably, the same viral proteins can also interact with TRIM-25 E3 ligase, which is essential for the ubiquitination of RIG-I and activation of the signaling cascade (Table S1). The SARS-CoV-2 proteins can also interact with effector targets of the RIG-I signaling like IRF3 and IRF7 and inhibit its translocation to the nucleus and downregulate IFN-I production [22]. We also observed that viral protein might interact with components of TLR-mediated IFN response like TRAF6 and IRAK1 [23].

**Fig. 2:**
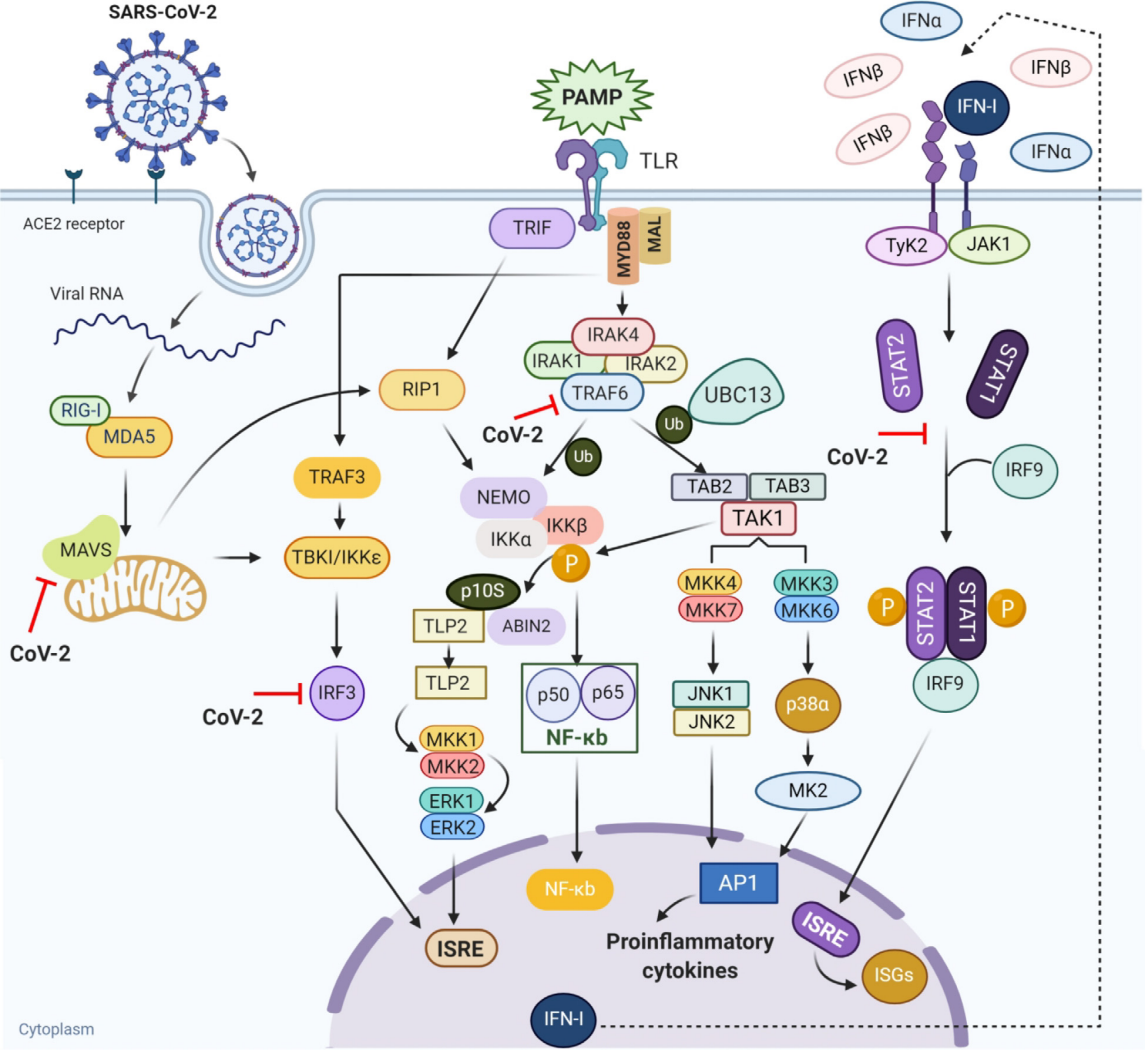
Type I interferon induction and signaling during SARS-CoV-2 infection and virus-mediated inhibition of IFNI and ISGs. The schematic diagram represents key players of INF-I pathways associated with SARS-CoV-2 infection. After entering the cells, the innate sensors such as MDA5, RIG1, and PAMS recognize the viral proteins and nucleic acids, which then activate IFN-I and proinflammatory cytokines production. Subsequently, INF-I enhances the production of ISGs and sets the stage for the potent antiviral immune response. Simultaneously, the SARS CoV-2 proteins interact with the MAVS, TRAF6, IRAK1 and negatively regulate IFN-I signaling and dampen host immune response. Abbreviations: TyK2: Tyrosine kinase 2, JAK1: Janus kinase 1, MK2: MAPK-activated protein kinase 2, MKK1/2/3/4/6/7: Mitogen-activated protein kinase, ERK1/2: extracellular signal-regulated kinases, JNK1/2: c-Jun N-terminal kinases, TAB2/3: TGF-Beta Activated Kinase Binding Protein, TAK1: TGF-beta-activated kinase, ABIN2: TNFAIP3 interacting protein 3, IRF 3/9: Interferon regulatory factor 3/ 9, STAT1/2: Signal Transducer and Activator Of Transcription, IKKα: IκB Kinase α, IKKβ: IκB Kinase β, NEMO: NF-Kappa-B essential modulator, RIGI: retinoic acid-inducible gene I, MDA5: melanoma differentiation-associated protein 5, MAVS: Mitochondrial antiviral-signaling protein, TBK1: TANK Binding Kinase 1, TRAF3: TNF Receptor Associated Factor 3, TRAF6: TNF Receptor Associated Factor 6, TRIF: TIR domain-containing adapter molecule 1, MYD88: Myeloid differentiation primary response protein MyD88,IRAK1/2/4: Interleukin-1 receptor-associated kinase, PAMP: Pathogen-associated molecular pattern, TLR: Toll-like receptors, TLP2: Thioredoxin, ISG: Interferon-stimulated gene, AP1: Activator protein 1, ISRE: Interferon-Stimulated Response Element, IFN-1: Type I Interferon, RIP1: Receptor Interacting Serine/Threonine Kinase 1. The figure was created with Biorender.com.

The IFN-I signaling can also activate mitogen-activated protein kinase (MAPK) signaling regulating several cellular processes ranging from inflammation, cell differentiation, stress to apoptosis, and metabolism. On the other hand, the MAPK signaling pathway is activated by several viruses. Interestingly, MAPK is essential for coronavirus replication and associated with the production of the inflammatory cytokines (Fig. 2) [24], [25]. In this context, the degree centrality analysis of the predicted 369 host genes revealed that the MAPK signaling pathway has a central role in the innate immune signaling response to SARS-CoV-2 infection. Downstream, the MAPK-signaling could trigger a cascade of other potent pathways, potentially influencing the outcome of viral pathogenesis [26].

### 3.4 TP53 and VAMP8 respectively are the key proteins of cytokines storm and neutrophil degranulation process associated with acute respiratory distress syndrome (ARDS)

**Fig. 3:**
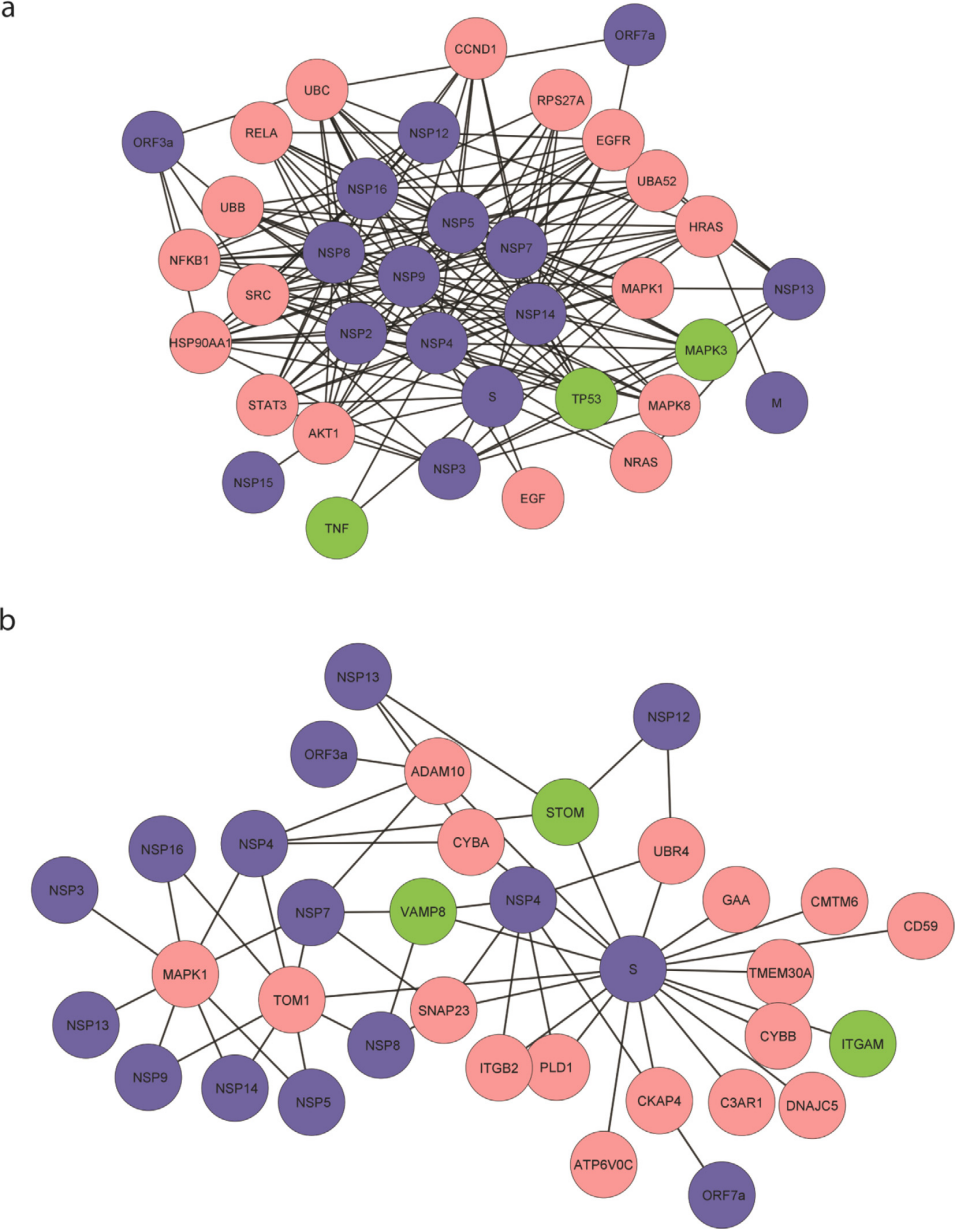
A predicted interaction map of SARS-CoV-2 proteins and top 20 host proteins associated with cytokine signaling pathway and Neutrophils degranulation. Blue color represents the virus proteins, and brown color represents the human interactor proteins. (a) Sixteen SARS-CoV-2 proteins interact with human proteins associated with cytokine pathway. TP53, TNF, and MAPK3 (represent in green color) are highly weighted proteins and may regulate the cytokine production. (b) Thirteen SARS-CoV-2 proteins interact with human proteins associated with Neutrophils degranulation. Degree centrality analysis reveals that VAMP8, ITGAM, and STOM (represented in green color) are the key players in neutrophils degranulation process. (For interpretation of the references to color in this figure legend, the reader is referred to the web version of this article.)

SARS-CoV-2 causes acute lung injury and acute respiratory distress syndrome (ARDS), which can result in severe lethal conditions [27]. Our results suggest that cytokine signaling and neutrophils degranulation are the most significant pathways associated with SARS-CoV-2 infection. We show that 299 candidates (associated with cytokine signaling) interact with SARS-CoV-2 proteins (Supplementary Table 3). The degree centrality analysis of these genes suggests that cellular tumor antigen p53 (TP53), followed by tumor necrosis factor (TNF), mitogen-activated protein kinase 3 (MAPK3), and mitogen-activated protein kinase 1 (MAPK1) have crucial and central roles in cytokine signaling (Fig. 3A) (Table 1). TNFα is a potent pro-inflammatory factor and a key regulator of immune cell functions. In viral infections, TNFα can induce pro-inflammatory cytokine production and activate TNF-dependent pathways. This primarily directs NF-κB-mediated cytokine production, ie; IL6, IL4 [28]. Also, SARS-CoV-2 viral proteins can stimulate the MAPK and NF-κB pathways (Fig. 3A), leading to the induction of IL6 production.

**Table 1:** Degree centrality analysis of proteins associated with Cytokines storm.

Serial Number	Host Protein	Ensemble Gene ID	Degree Centrality (Weight)
1	TP53	ENSP00000269305	114.272
2	TNF	ENSP00000398698	113.552
3	MAPK3	ENSP00000263025	110.762
4	MAPK1	ENSP00000215832	106.453
5	AKT1	ENSP00000451828	103.622
6	HRAS	ENSP00000407586	98.912
7	STAT3	ENSP00000264657	98.127
8	SRC	ENSP00000362680	98.033
9	RELA	ENSP00000384273	87.107
10	UBC	ENSP00000441543	84.869
11	NFKB1	ENSP00000226574	84.853
12	NRAS	ENSP00000358548	83.778
13	UBA52	ENSP00000388107	83.556
14	RPS27A	ENSP00000272317	82.657
15	UBB	ENSP00000304697	82.254
16	EGFR	ENSP00000275493	82.104
17	MAPK8	ENSP00000378974	79.934
18	HSP90AA1	ENSP00000335153	79.78
19	EGF	ENSP00000265171	79.206
20	CCND1	ENSP00000227507	78.367

Neutrophils are present during many lung diseases associated with acute respiratory distress syndrome (ARDS) and may be involved in severe lung injury [29] in COVID-19 patients. Neutrophils form extracellular web-like structures of DNA and proteins called neutrophil extracellular trap (NET). These NETs induce mucus accumulation in patients' airways and facilitate ARDS during viral infection [30]. Presently, we noticed that 193 proteins are associated with neutrophils degranulation and interact with SARS-CoV-2 proteins (Supplementary File 4). Degree centrality analysis suggests that vesicle-associated membrane protein 8 (VAMP8), integrin alpha-M (ITGAM), and erythrocyte band 7 integral membrane protein (STOM) are the key regulators of neutrophil degranulation (Fig. 3B) (Table 2). VAMP8 is a member of the soluble *N*-ethylmaleimide-sensitive fusion protein attachment protein receptor (SNARE) family of fusion proteins. It confines to secretory granules, and degranulation is inhibited in VAMP8-deficient macrophages and mast cells [31]. VAMP8 is also expressed in human neutrophils and may follow a similar mechanism of degranulation [32]. The complement system also activates neutrophils degranulation. The C5a triggers the phagocytic NADPH-oxidative burst and enhances phagocytosis and the release of granule molecules from neutrophils and macrophages [33], [34].

**Table 2:** Degree centrality analysis of proteins associated with neutrophil degranulation.

Serial Number	Host Protein	Ensemble Gene ID	Degree Centrality (Weight)
1	VAMP8	ENSP00000263864	72.583
2	ITGAM	ENSP00000441691	64.175
3	STOM	ENSP00000286713	62.88
4	C3AR1	ENSP00000302079	60.08
5	ITGB2	ENSP00000380948	58.371
6	DNAJC5	ENSP00000354111	57.133
7	TOM1	ENSP00000413697	56.9
8	CD59	ENSP00000379191	56.825
9	MAPK1	ENSP00000215832	56.629
10	CKAP4	ENSP00000367265	56.388
11	SNAP23	ENSP00000249647	54.835
12	CYBB	ENSP00000367851	54.389
13	TMEM30A	ENSP00000230461	54.252
14	CMTM6	ENSP00000205636	54.165
15	GAA	ENSP00000305692	52.909
16	ATP6V0C	ENSP00000329757	52.449
17	ADAM10	ENSP00000260408	52.051
18	PLD1	ENSP00000342793	51.552
19	UBR4	ENSP00000364403	50.564
20	CYBA	ENSP00000261623	50.427

### 3.5 The complement system-induced thrombosis in SARS CoV-2 patients

Extra-pulmonary microvascular injury associated with blood thickening and clotting is being reported in different organs and blood vessels of SARS-CoV-2 infected patients. These virus-induced thrombotic changes manifest in endotheliitis, and intravascular coagulation is presumed to be the major cause of stroke and other complications in patients. Earlier, Margo et al. published a case study on five seriously ill SARS CoV-2 infected patients and reported that complement components C3, C4, the terminal complex C5b-9 (also known as the membrane attack complex (MAC)) were deposited on the coagulation area in the lungs alveoli [35]. The COVID-19 patients showed thrombocytopenia and possibly elevated D-dimer level, a fibrinolysis-specific degradation product [36], [37]. The thrombocytopenia and elevated D-dimer can be described by the extreme activation of the coagulation cascade and platelets [38].

**Fig. 4:**
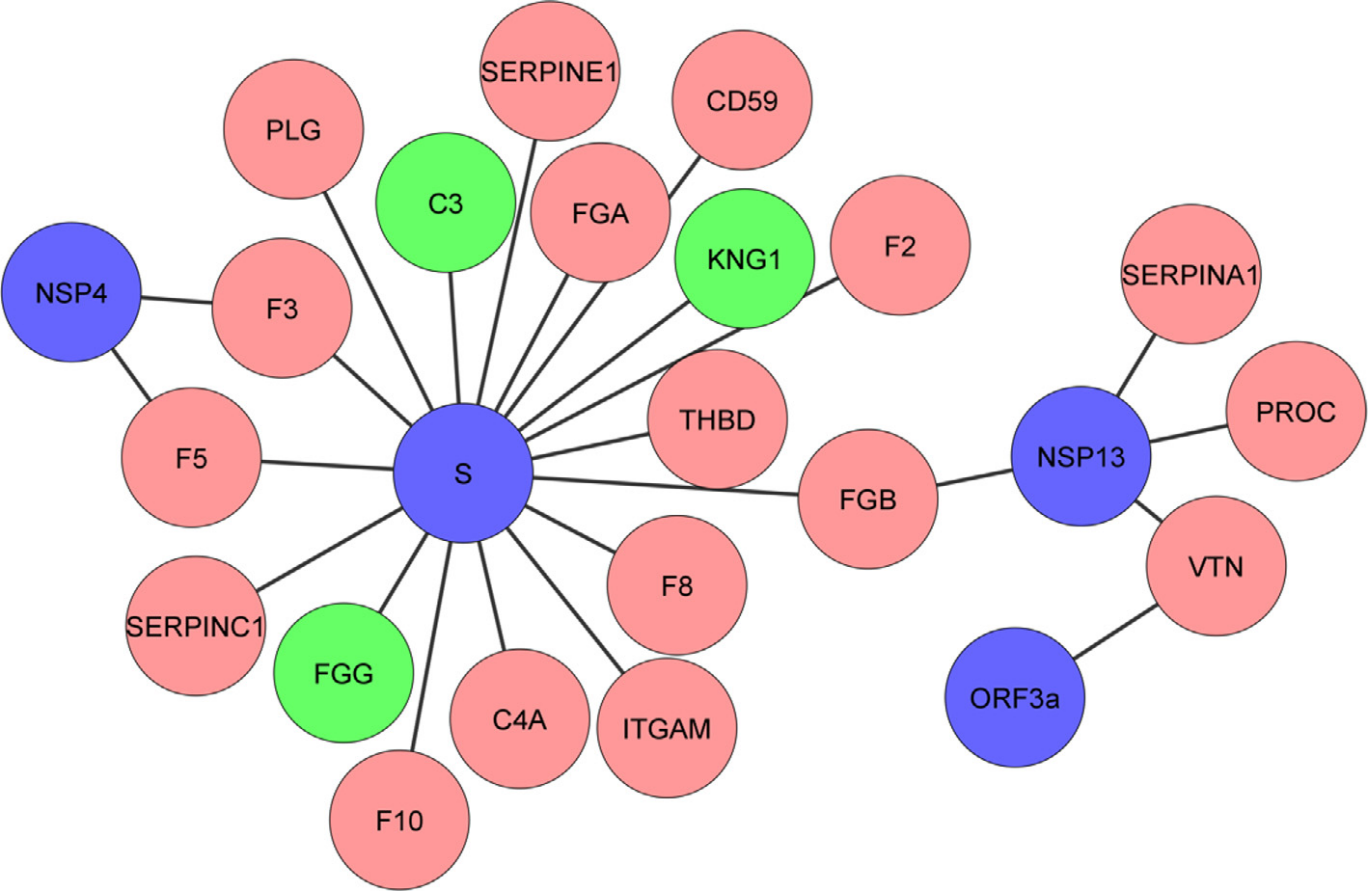
A predicted interaction map of SARS-CoV-2 proteins and the top 20 host proteins associated with complement and coagulation cascade. Blue color represents the virus proteins, and brown color represents the human interactor proteins. Four SARS-CoV-2 proteins interact with human proteins associated with complementing and coagulation cascade. Most of the proteins interact with spike protein. Degree centrality analysis reveals that KNG1, C3, and FGG (represented in green color) are the key players in complement and coagulation cascade. (For interpretation of the references to color in this figure legend, the reader is referred to the web version of this article.)

In our KEGG pathway and gene enrichment analysis, we discovered two highly enriched, prothrombotic, i.e., Complement and coagulation cascade, and platelet activation pathways, presumably directing thrombotic activities in severe COVID-19 patients. Our analysis shows that 52 proteins related to Complement and coagulation cascades interact with SARS-CoV-2 proteins (Supplementary Table 3). Degree centrality analysis of these genes suggests that Kininogen-1 (KNG1), followed by C3, and Fibrinogen gamma chain (FGG), play a crucial role in activating Complement and coagulation cascades (Fig. 4) (Table 3). Our study suggests that among all SARS-CoV-2 proteins, the spike protein showed high weighted interaction with proteins associated with complement and coagulation cascade and could activate proteolytic processing of C3. The C3 hydrolysis leads to C3a and C3b, and subsequently further cleavage of C5 into C5a and C5b. Activation and generation of C3a and C5a are well-known inducers for the production of inflammatory cytokines that leads to tissue damage in the lungs.

**Table 3:** Degree centrality analysis of proteins associated with complement and coagulation cascade.

Serial Number	Host Protein	Ensemble Gene ID	Degree Centrality (Weight)
1	KNG1	ENSP00000265023	30.174
2	C3	ENSP00000245907	29.864
3	FGG	ENSP00000336829	29.062
4	FGA	ENSP00000306361	28.534
5	PLG	ENSP00000308938	28.522
6	SERPINC1	ENSP00000356671	28.022
7	F2	ENSP00000308541	27.394
8	FGB	ENSP00000306099	25.675
9	PROC	ENSP00000234071	23.989
10	F5	ENSP00000356771	23.368
11	C4A	ENSP00000396688	22.361
12	F3	ENSP00000334145	22.34
13	SERPINE1	ENSP00000223095	21.536
14	F8	ENSP00000353393	21.432
15	F10	ENSP00000364709	20.901
16	SERPINA1	ENSP00000416066	20.644
17	THBD	ENSP00000366307	20.425
18	CD59	ENSP00000379191	18.566
19	ITGAM	ENSP00000441691	18.562
20	VTN	ENSP00000226218	17.758

**Fig. 5:**
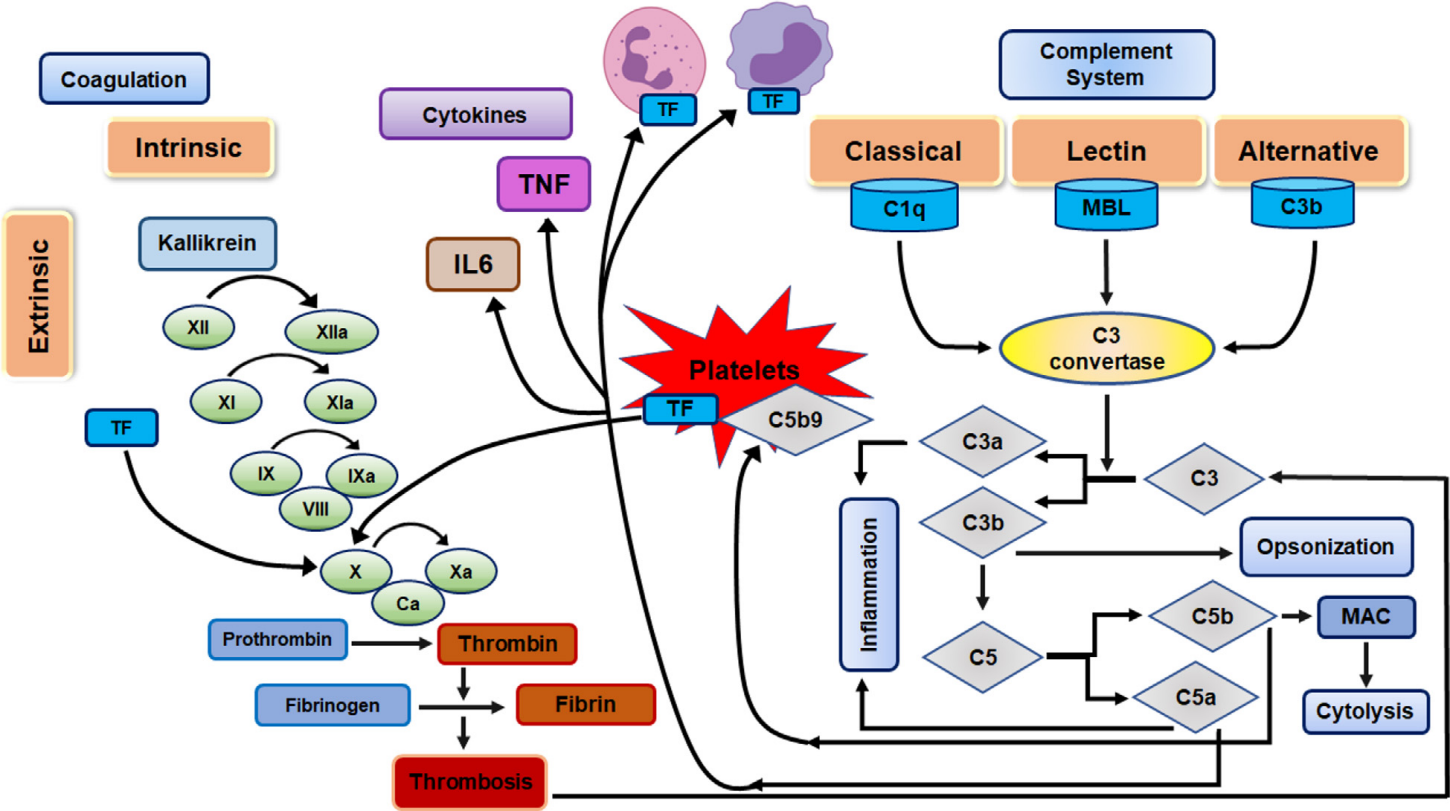
Schematic representation of the mechanism of complement system-mediated thrombosis. SARS-CoV-2- Spike (S) protein interacts with C3 and activates proteolytic processing of C3, C3 hydrolysiszes in C3a and C3b, and further C3b cleaves C5 into C5a and C5b. C3a and C5a induce inflammatory cytokine production. Further, C3a, C5a, and C5b9 activate the coagulation pathway. C5a also activates IL6 and TNF alpha production, which magnifies the coagulation pathway.

The alteration of cellular membranes is essential in platelet activation, which is required for the primary clot formation. Association of the complement C5b-9 complex into the cell membrane activates platelets and results in the appearance of procoagulant lipids [39]. Modification of the phospholipid is also required for the tissue factor (TF)-based coagulation pathway. TF starts the extrinsic pathway of coagulation and is highly expressed in various tissues, including the brain, lung, kidney, and placenta [40]. Our analysis showed that the activated complement substrate, specifically C5, can cause an increase in the expression of functionally active TF in leukocytes and endothelial cells (Fig. 5) [41]. Although neutrophils carry TF on their membranes, it is unclear whether they can produce TF alone. It is tempting to speculate that the enhanced coagulation process observed in SARS-CoV-2 infection is due to higher neutrophils degranulation.

### 3.6 Genes associated with complement and coagulation pathways are upregulated in the SARS-CoV-2 infected Huh7 cells

**Fig. 6:**
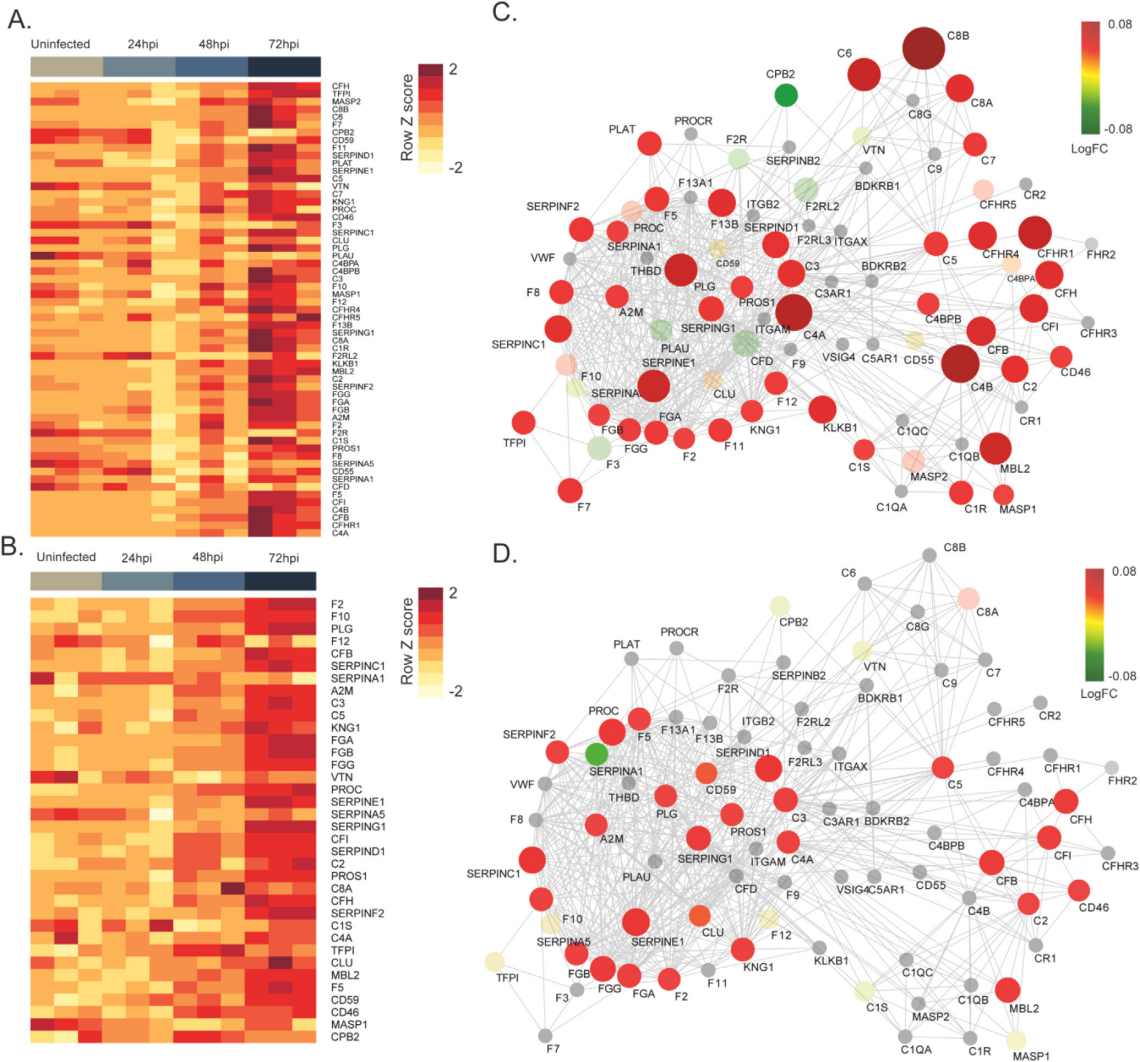
*In vitro* validation of the complement and coagulation cascade in Huh7 cells. (a) Heatmap of 59 genes associated with the KEGG pathway “Complement and coagulation cascades” that were detected in the transcriptomics over the indicated time of infection. (b) Heatmap of 36 proteins associated with KEGG pathway “Complement and coagulation cascades” that were detected in the proteomics data over the indicated time of infection. Data were quantile normalized, and Z-score transformed. Lower values are represented in yellow and higher values in red. (c) Cytoscape network of KEGG pathway “Complement and coagulation cascades” labeled with transcriptomics results. 59 transcripts were detected and 44 have differential expression over time. (d) Cytoscape network of KEGG pathway “Complement and coagulation cascades” labeled with proteomics results. 36 proteins were detected and 28 have differential abundance over time . Genes or proteins are represented as circles. Gradient color was applied to proteins depending on temporal fold change calculated by LIMMA (low = green to high = red). Nonsignificant proteins or transcripts are represented with transparency. Non detected proteins are represented grey. The size of the significant protein is proportional to the fold change. (For interpretation of the references to color in this figure legend, the reader is referred to the web version of this article.)

Our *in silico* analysis showed strong evidence of complement and coagulation cascade in SARS-CoV-2 infection. We then validated our findings in *in vitro* experiments by re-analyzing the proteomics and transcriptomics data from the study by Appelberg et al., where Huh7 cells were infected with SARS-CoV-2 over a period of 72 h. The proteomics and transcriptomics data sets were analyzed against 85 genes associated with the ‘Complement and coagulation pathways’ obtained from the KEGG database. Globally, there was a constant increase in transcript expression (Fig. 6A) and protein abundance (Fig. 6B) of coagulation associated genes from the uninfected stage to 72 h post-infection. Fold change of hits was relatively low because proteins or transcript abundance were not increasing constantly. In the main study by Appelberg *et al.,* the authors showed that the most significant increase in transcriptomics was between 48 and 72 h while for proteomics, it was between 24 and 48 h. The network of experimentally observed genes associated with complement and coagulation cascade shows a highly interconnected network (Fig. 6 C, D). The network was annotated with transcriptomics (Fig. 6C) and proteomics data (Fig. 6D). Among 85 genes associated with complement and coagulation, 59 transcripts were detected, of which 44 showed significant changes in the transcriptomics data set. While in the proteomics data set, 36 proteins were detected, and 28 showed significant changes (Supplementary Table 5). There was a considerable overlap of 24 genes/proteins as shown in supplementary Fig. 3. Thus, by analyzing both (*in silico* and *in vitro*) data sets, we suggest that most proteins and transcripts are up-regulated during the course of infection and strongly activate the complement and coagulation pathways. These findings can be extrapolated to existing pathophysiological observations of thrombotic events in COVID-19 disease, which could be directly influenced by activation of complement and coagulation pathways.

## 4 Discussion

In the study, we established a network of protein–protein interactions between the host and SARS-CoV-2 proteins by using a protein structure-based computational approach. The GO analysis of these proteins identified their involvement primarily in the innate immune system, cytokine signaling, MAPK signaling pathway, neutrophils degranulation, complement, and coagulation cascades. On further analysis, we discovered that all these pathways overlap considerably with a key set of highly connected genes. The degree centrality analysis revealed that MAPK1, MAPK3, AKT1, and SRC genes are crucial for these pathways and overlapped maximally. This information suggests the existence of a nexus of robust inflammatory events upon SARS-CoV-2 infection. As MAPKs are heterogeneous kinases capable of phosphorylation of serine and threonine residues present in many proteins, the emergence of MAPK-associated pathways are seen as the likely reason for a profound pro-inflammatory cytokines production: stimulation and induction of strong innate response in the host. We also discovered that to counter IFN-I, a few SARS-CoV-2 proteins may interact with IFN-I signaling pathway associated proteins like MAVS, IRF3, TRIM21, TRAF6, and IRK1 and could inhibit their functions [23], [42]. By combining these observations, we suggest that the MAPK signaling pathway is central to the innate immune response to SARS-CoV-2 infection and associated pathophysiology. We have also found some other enriched pathways, which may be crucial for SARS-CoV-2 mediated pathogenesis, such as PD-L1 expression and PD-1 checkpoint pathway in cancer and C-type lectin receptor signaling pathways. A recent clinical study shows that PD-L1 expression is associated with COVID-19 patient severity. It is highly activated in COVID-19 severe patients [43]. and it was reported earlier that PD-1 and PD-L1 are vital mediators in T cell depletion in sepsis and cancer patients [44], [45]. Our observations are in line with clinically approved drug Camrelizumab (human anti-programmed cell death 1 (PD-1) monoclonal antibody) with thymosin which is already in use against SARS-CoV-2 [46]. Similarly, C-type lectin receptor signaling pathways are also enriched in our study and might be associated with the induction of the proinflammatory response after virus infection [47].

Clinical studies and case reports suggest the COVID-19 disease severity is associated with the degree of ARDS, lung injury, hypoxia, thrombosis, ischemia, and reperfusion injury. These pathological manifestations are also observed along with the increased cytokine storm, neutrophils degranulation, and activation of components of the complement and coagulation pathway [48], [49]. We show highly enriched pathways supporting these clinical observations and further unravel the plausible mechanism behind these phenomena. The degree centrality analysis shows cytokine-associated pathways are regulated by key players such as TP53, TNF, MAPK3, and MAPK1 proteins. Similarly, degree centrality analysis of neutrophils degranulation genes highlighted VAMP8, ITGM, and STOM to be the highly weighted proteins in this pathway. Thus, we propose that these molecules could be the target to mitigate viral infection.

Most strikingly, our observations were narrowed down to the Complement and Coagulation cascade's activation and showed their interconnectivity in SARS-CoV-2 pathogenesis. These findings could be a step forward in solving the thrombotic events observed in the severe cases of COVID19 patients [50]. Our *in vitro* experimental results identified a vast majority of known players associated with complement and coagulation during the course of viral infection. On the other hand, the complement system also inhibits an anticoagulation process. The C4b-binding protein (C4BP) is an essential cofactor for the enzymatic degradation of C4b. It can make a complex with protein S (PS), a vitamin-K dependent anticoagulant glycoprotein. The PS is the cofactor of activated protein C (APC), which degrades coagulation factors Va and VIIIa. Formation of the PS–C4BP complex results in a loss of PS cofactor activity, thereby reducing its anticoagulant effects [51]. After proteolytic cleavage of complement cascade results in the formation of C3a and C5a, which regulate cytokine response and enhance the production of TNF and IL6 [52]. Cytokines like TNF and IL6 also are strongly associated with the coagulation process. TNF is a potent enhancer of TF expression on monocytes, and IL-6 enhances the production and thrombogenicity of platelets [53]. Therefore, they can also decrease the anticoagulant factors like thrombomodulin, the endothelial cell protein C receptor, and PS [53]. Activation of the complement system, coagulation cascade, and neutrophiles degranulation are interconnected and could result in the pleiotropic effect in response to the SARS-CoV-2 infection. This could exacerbate pathogenesis of lung parenchymal cells, diminish oxygen uptake, and cause rapid thrombotic events and intravascular coagulation, which could result in multiorgan failure including central nervous system complications and death [54].

Among the viral proteins, spike protein interacts with complement components such as with C3, C4A, C5, and ORF 3a product interacts with mannose-binding lectin2 (MBL2). This observation is in line with earlier studies conducted on SARS-CoV-1, which revealed a direct interaction with MBL2 and activation of the complement system [55], [56]. The C3 is a key regulatory protein of the complement system. This notion is supported by a recent study on SARS-CoV-2, which revealed that activation of complement component C3 exacerbates disease pathology in SARS-CoV associated ARDS. Mice deficient with C3 (C3^−/−^) exhibited significantly lower respiratory dysfunction despite having equivalent viral loads in the lungs. This was associated with significantly fewer neutrophils and inflammatory monocytes present in the lungs of C3^−/−^ compared to the control mice. Subsequent investigations exhibited reduced lung pathology and lower cytokine and chemokine levels in the tissue and the sera of C3^−/−^ mice [57]. Thus, the Complement system chemoattractant property may cause neutrophilia in damaged tissue. Based on previous studies and our experimental results, we propose the complement system, along with neutrophils, significantly modulate the thrombogenesis pathway during SARS-CoV-2 infection.

Complement system plays a crucial role during the entire course of pathological events during COVID-19 disease progression. A recent study shows that anti-C3 inhibitor AMY-101 successfully treats COVID-19 patients [58]. This supports our findings that the complement system is a key pathway of the innate immune system associated with SARS-CoV-2 mediated pathogenesis [59]. We propose active research on antiviral drug discovery front targeting the key molecules on the complement and coagulation cascade pathway. Our study is limited to *in silico* and *in vitro* experiments and warrants further detailed clinical studies including samples obtained from critically ill COVID-19 patients. Further, future studies with animal models with genetically modified hosts could also be helpful in unraveling the exact mechanism of this phenomenon.

## 5 Author’s contributions

RT, ARM, DN conceptualized the study. RT, ARM performed data mining, bioinformatics studies, and computational biological work. FM and SG conducted *in vitro* experiments and analyzed the data. AM supported in virus infection assays. SNB edited the manuscript and involved in ideas generation. UN supervised *in vitro* experiments, edited manuscript. RT, ARM and DN wrote manuscript.

## Declaration of Competing Interest

The authors declare that they have no known competing financial interests or personal relationships that could have appeared to influence the work reported in this paper.
