# Supplementary material for: In silico and in vitro studies reveal complement system drives coagulation cascade in SARS-CoV-2 pathogenesis
Source: Comput Struct Biotechnol J. 2020 Nov 11;18:3734–44. doi: 10.1016/j.csbj.2020.11.005 (PMC7657020; doi:10.1016/j.csbj.2020.11.005)
Supplement: Supplementary data 1 [file mmc1.docx]

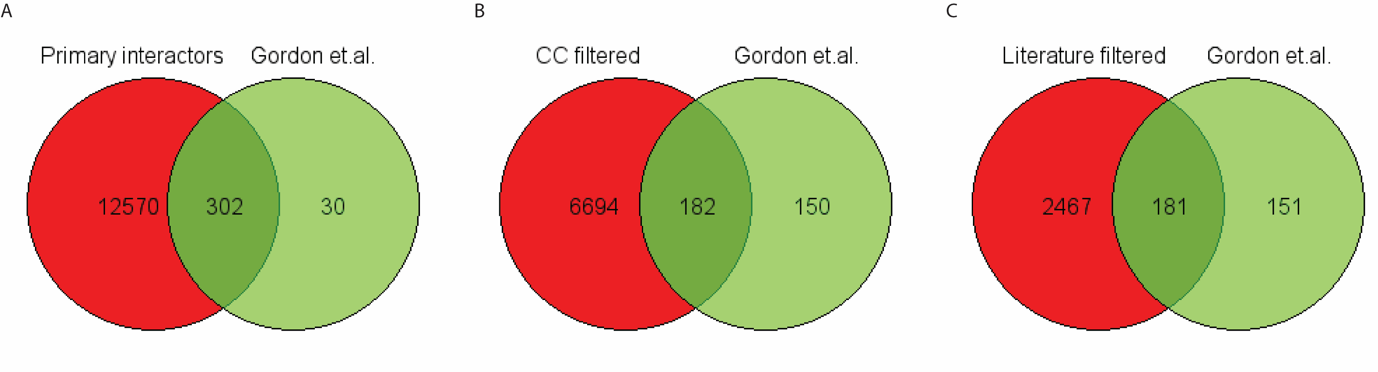


**Figure S1: Overlap with previous studies:**

(A) Figure represents the overlap proteins between primary interactors list and that of list published by Gordon et.al.. Note ~91 % of the list provided in Gordon et al study overlap with the candiates predicted in our study. (B,C) Diagram represent the overlap proteins between CC and literature filtered protein with Gordon et al. proteins.


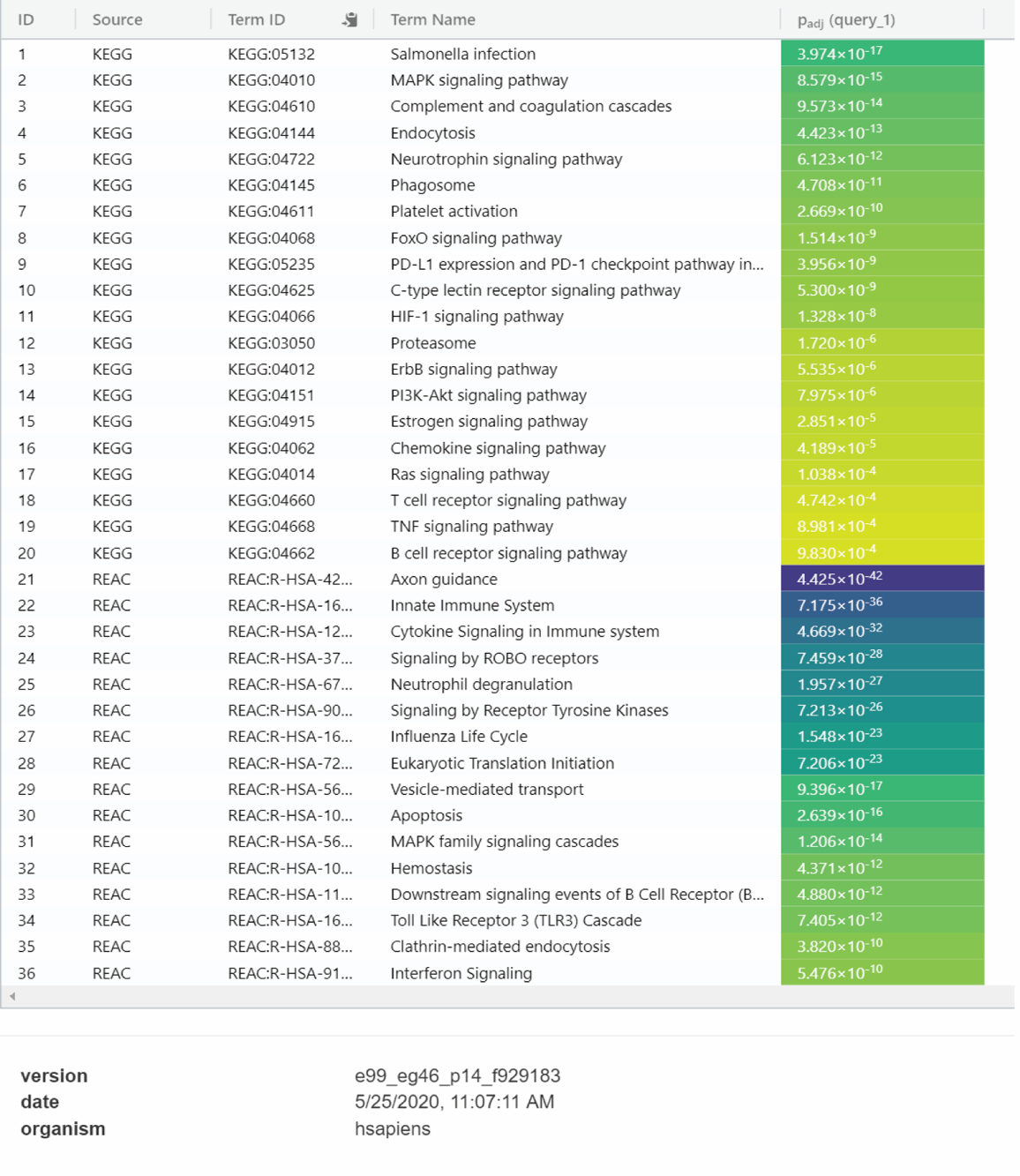


**Figure S2: List of significantly enriched KEGG terms and Reactome enhanced data**

Compilation of 2,647 unique proteins entries resulting in highly enriched biomedical relevant KEGG term and the Reactome pathways listed above having p-adjusted p-values.


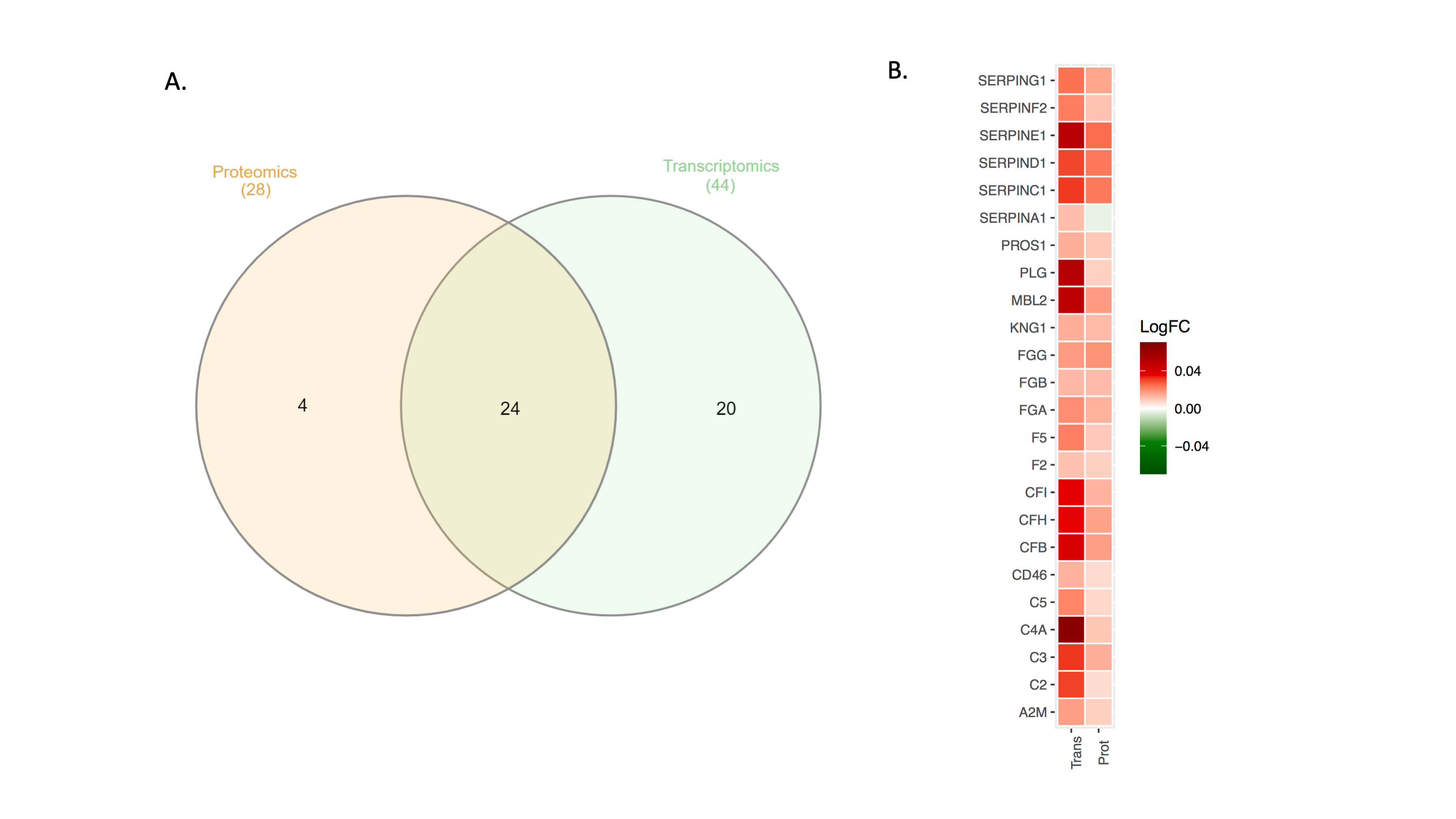


**Figure S3: Venn diagram represents the overlap between transcripts and proteomics**

**data**

**(**A) Venn diagram showing the overlap between transcripts (RNAseq) and proteins (LC-MS/MS) associated with KEGG term complement and coagulation cascades and evolving significantly with covid infection over time. (B) Heatmap representing LogFC of genes associated with KEGG term complement and coagulation cascades and evolving significantly with SARS-CoV-2 infection over time in both the proteomics and transcriptomics data.
